# Effect of Power Output and Pedaling Cadence on Plantar Pressures in Professional Cyclists with Overuse Injuries

**DOI:** 10.3390/sports14050184

**Published:** 2026-05-06

**Authors:** Dídac Navarro-Martínez, Javier Zahonero, Pablo Vera, José Martí-Martí, Florentino Huertas, Carlos Barrios

**Affiliations:** 1 Department of Physical Preparation and Conditioning, Faculty of Physical Activity and Sport Sciences, Catholic University of Valencia, 46900 Torrent, Spain; didac.navarro@ucv.es (D.N.-M.); jose.marti@ucv.es (J.M.-M.); florentino.huertas@ucv.es (F.H.); 2 Institute for Research on Musculoskeletal Disorders, School of Medicine and Health Sciences, Catholic University of Valencia, 46001 Valencia, Spain; fp.vera@ucv.es (P.V.); carlos.barrios@ucv.es (C.B.)

**Keywords:** cycling biomechanics, plantar pressure, pedaling technique, power output, cadence, overuse injuries

## Abstract

**Background**: Plantar pressure analysis provides insight into load distribution at the foot–pedal interface during cycling; however, its modulation by pedaling power, cadence, and overuse injury status remains poorly understood by professional cyclists. It is unclear whether common overuse injuries, such as Achilles tendinopathy, patellofemoral pathology, and iliotibial band syndrome, are associated with distinct plantar loading patterns. This study aimed to characterize plantar pressure distribution in elite cyclists and determine how power, cadence, and injury status influence this pattern. **Methods**: Professional cyclists completed a single integrated protocol using a high-resolution in-shoe pressure system. Plantar forces were recorded across nine anatomical regions and grouped into the transverse and longitudinal segments of the foot. Three phases were included: absolute power manipulation (100 and 200 W), cadence manipulation (80 and 100 rpm) at fixed power, and an ecological combined protocol using relative power (1.5 and 3 W·kg^−1^) with individualized cadence. The cyclists used their habitual bike setups. Participants were classified into the non-pathological (NP), AT, PFP, or ITBS groups. Mixed repeated-measures ANOVAs were used to analyze the effects of power, cadence, zone, foot, and injury status. **Results**: The plantar pressure distribution was consistently dominated by the medial forefoot. Increasing the absolute power from 100 to 200 W increased the maximal plantar pressures by 84.74% (*p* < 0.001), whereas increasing the cadence from 80 to 100 rpm at a fixed power increased the pressures by 15.90% (*p* = 0.003). Under individualized conditions, increasing relative power from 1.5 to 3 W·kg^−1^ increased pressures by 39.59% (*p* < 0.001), whereas cadence had no global main effect but significantly altered the regional pressure distribution (*p* < 0.001). Injury groups showed pathology-specific deviations, including higher overall pressures and asymmetry in Achilles tendinopathy, bilateral asymmetry in patellofemoral pathology, and asymmetric loading patterns in iliotibial band syndrome. **Conclusions**: Power is the main determinant of plantar pressure, and cadence modulates load distribution. Overuse injuries induce pathology-specific pressure patterns, supporting plantar pressure analysis for injury prevention and performance optimization in athletes.

## 1. Introduction

The transfer of force from the lower limb to the bicycle occurs through the shoe–pedal interface, where repetitive pedaling mechanics and constrained joint motion expose professional cyclists to a high risk of overuse injury. Although posture, technique, and joint kinetics in cycling have been extensively examined, plantar pressure, the final expression of load transmission through the kinetic chain, has received comparatively little attention. Power output is the most accurate indicator of exercise intensity [1], and increases in power alter muscle activation [2], knee joint forces [3], and the magnitude of effective pedal forces [4,5], leading to higher plantar pressures, particularly under the first metatarsal head and hallux [5]. Previous studies have been largely descriptive [5,6] or have focused on footwear interventions without addressing injury status [7].

Cadence modulates pedal forces [8] and plantar pressure [5,9]. Higher cadences reduce pressure magnitude, particularly in the forefoot, although the optimal cadence remains debatable [10], and cyclists generally self-select cadence based on task demands [11]. Because cadence influences muscle recruitment [12,13], fiber utilization [14], and mechanical loading, it may also shape plantar loading; however, this relationship has not been well-documented [5].

Plantar pressure has been associated with Achilles tendinopathy [15], patellofemoral pain [16], and iliotibial band syndrome [17], although these findings come from running and walking, whose loading patterns differ substantially from cycling. Moreover, most cycling studies rely on absolute workloads, despite the clear influence of body mass on mechanical demand [18]. Relative power (W/kg) is a more meaningful metric [19], and cadence should be individualized rather than being prescribed at fixed values.

Despite these considerations, no study has examined how absolute power, absolute cadence, and individualized combinations of relative power and individualized cadence influence plantar pressure distribution in professional cyclists or how these patterns differ across common overuse injuries.

To address this gap, the present study quantified plantar pressures across these three controlled pedaling conditions and compared loading patterns between non-pathological cyclists and those with typical overuse injuries. The purpose of this study was to determine how pedaling power and cadence independently and jointly influence plantar pressure distribution, and to identify whether injury-specific patterns emerge under different loading conditions.

## 2. Methods

### 2.1. Study Design

This study employed a controlled within-subject experimental design to investigate how variations in pedaling power and cadence influence plantar pressure distribution in professional cyclists, with three predefined primary outcomes: the effect of increasing power at a fixed cadence, the effect of increasing cadence at a fixed power, and the combined effect of individualized relative power (expressed as W/kg) and individualized cadence. The protocol was structured as a unified experimental framework performed on separate days, and plantar pressure patterns were compared between cyclists with common overuse injuries and those without pathology.

### 2.2. Sample Size Calculation

A priori sample size calculation for the primary Group × Condition interaction was conducted using G*Power 3.1.9.7. A mixed repeated-measures ANOVA (within–between interaction) was specified with two groups and two repeated measurements. Based on a medium expected effect size (f = 0.25), α = 0.05, desired power 1 − β = 0.80, an assumed correlation of 0.50 between repeated measures, and ε = 1 (required for two measurements), the required total sample size was 34. This value corresponds to approximately 17 cyclists per group to achieve 80% power to detect the hypothesized interaction effect. This analysis was intended to support the main planned repeated-measures comparison underlying the study design rather than the full multi-factorial ANOVA models, including multiple within-subject factors (e.g., foot and plantar region) and separate injury subgroup analyses. Therefore, although the total sample was adequate for the principal repeated-measures comparisons, analyses involving specific injury subgroups should be interpreted with caution because these comparisons may have been underpowered, particularly in smaller pathology groups.

### 2.3. Participants

Professional cyclists were recruited from the WorldTour and UCI Pro-Continental road teams and the Spanish national track program. A total of 50 male cyclists participated in the absolute-load conditions, and 49 cyclists (43 men and 6 women) completed the individualized combined protocol. Because only six women were included, sex was not entered as a covariate and no sex-stratified analyses were performed; therefore, female data in the individualized protocol were pooled with those of the male participants. All participants met the criterion of trained cyclists described by Jeukendrup [20], which was selected because of the difficulty of interpreting results when mixing elite-trained and recreational cyclists in biomechanical research. Injury assessment was performed clinically by a physiotherapist and a podiatrist, and cyclists were classified as non-pathological (NP) or as presenting with Achilles tendinopathy (AT), patellofemoral pathology (PFP), iliotibial band syndrome (ITBS), or other overuse injuries (OT). The OT category comprised clinically identified overuse conditions other than AT, PFP, or ITBS, as determined by the evaluating clinicians, and none of these conditions prevented participation in training or testing. Some cyclists presented more than one injury, which explains why the total number of pathological classifications exceeded the total number of participants in Table 1. All the cyclists provided written informed consent prior to participation. Eight participants presented with more than one overuse injury and were therefore included in multiple pathology-specific categories. Specifically, these participants contributed to more than one injury classification (e.g., AT and PFP), resulting in a non-mutually exclusive subgroup membership. Consequently, the injury groups were not statistically independent, and comparisons between the pathology-specific groups and the NP group should be interpreted with caution.

### 2.4. Inclusion and Exclusion Criteria

Cyclists were eligible if they were active professionals who either did not present any overuse injury or presented with a diagnosed overuse injury that did not impair their ability to train, compete, or complete the experimental protocol. Cyclists were excluded if they were unable to complete all experimental conditions or if their plantar pressure recordings were unusable owing to abnormal sensor behavior or incompatible insole configuration. Four cyclists were removed from the individualized condition because the newly replaced Biofoot/IBV^®^ insoles, although identical in size, displayed sensor layouts that could not be configured to match the criteria required for analysis.

### 2.5. Instrumentation

Plantar pressures were recorded using the Biofoot/IBV^®^ system (Instituto de Biomecánica de Valencia, Valencia, Spain), a validated mobile in-shoe pressure measurement instrument [21] that has been used in several peer-reviewed publications. The insoles included 62–64 piezoelectric sensors (depending on foot size), each 0.5 mm thick and 5 mm in diameter, and transmitted data telemetrically to a laptop via a transmitter worn at the waist of the participant. The Biofoot/IBV^®^ was sampled at 250 Hz while recording both feet simultaneously. Previous validation studies have reported excellent reliability comparable to that of the Pedar^®^ instrumented insoles [22].

**Figure 1:**
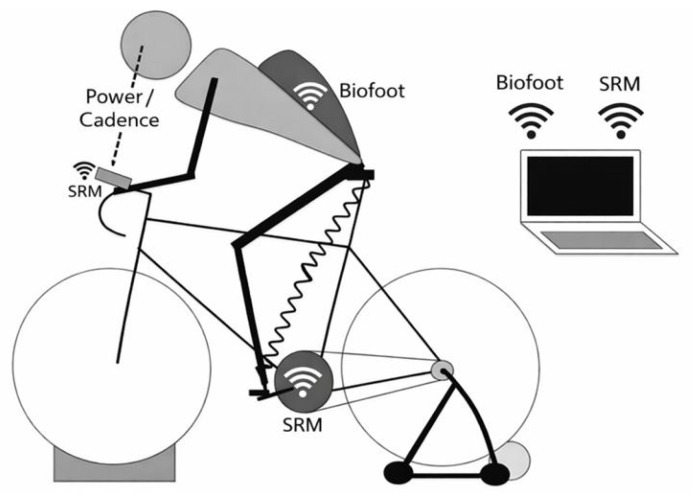
Configuration of the data collection system.

Power and cadence were measured using an SRM^®^ crank-based power meter (Schoberer Rad Messtechnik, Jülich, Germany). The SRM^®^ system is widely recognized as one of the most accurate devices for power assessment, with minimal errors in power measurement [23]. Its validity was confirmed by Martin, Milliken, Cobb, McFadden, and Coggan (1998) [24], who reported a 2.36% difference compared with the Monark^®^ 818 ergometer (r^2^ = 0.99). Owing to this level of validity, the SRM^®^ system has been used as the criterion device for validating newer power meters such as PowerTap^®^ [25], Look Keo Power^®^ [26], Axiom Power Train [25], and Ergomo^®^ Pro [27]. The cyclists used their own bicycles and shoes, with their normal individualized bike settings, mounted on an ELITE^®^ trainer (Elite Srl SB, Fontaniva, Italy) with a front-wheel leveling block, as shown in Figure 1. Accordingly, pedal and cleat systems were not standardized across participants, and cyclists used their habitual automatic binding systems (e.g., Shimano SPD-SL, Look Keo, Speedplay, or equivalent systems). This approach was chosen to preserve ecological validity and reflect real-world professional cycling conditions; however, the pedal system type was not controlled as an experimental factor. The cycling shoe type was not standardized or formally recorded as an analytical variable. All participants used their habitual competition shoes during the testing to preserve ecological validity. The cleat position and angular float were not standardized or formally recorded as analytical variables. Participants used their habitual cleat settings during testing as part of an individualized competition setup.

### 2.6. Experimental Protocol

All sessions were conducted in a controlled laboratory environment (23–25 °C) between 10:00 and 12:00. The cyclists avoided heavy training the day before and did not complete it in the preceding seven days. A physiotherapist and podiatrist conducted anthropometric measurements and injury anamnesis prior to testing. The SRM^®^ system was calibrated according to the manufacturer’s instructions, the Biofoot/IBV^®^ insoles were fitted and checked for wrinkles, pre-warmed by 3sds of bipedal standing followed by 10–1 walking steps and then calibrated per manufacturer guidelines.

The full experimental study consisted of three condition blocks performed on different days but was treated as a single, integrated experimental protocol. The first block examined the effects of absolute power. The cyclists completed a 10 min warm-up at 80–100 W and 80–90 rpm, followed by 3 min of rest. Two power conditions were then performed at a fixed cadence of 100 rpm: 100 W and 200 W. Each condition included 30 s of stabilization to ensure steady pedaling, followed by a 6 s plantar-pressure recording. At 100 rpm, this recording window captured approximately 10 complete pedal revolutions per condition, which was considered sufficient to characterize the steady-state plantar pressure pattern after the stabilization period. The conditions were separated by 3 min of low-intensity pedaling and were counterbalanced across cyclists to avoid fatigue or learning effects. Invalid sensor readings resulted in the exclusion of certain trials.

The second block evaluated the effect of the absolute cadence. The preparation was identical to that of the power block, with power fixed at 100 W and cadence manipulated between 80 rpm and 100 rpm. Each condition included 30 s of stabilization and a 6 s recording, with 3 min rest periods and counterbalanced order. This corresponded to approximately 8 pedal revolutions at 80 rpm and 10 pedal revolutions at 100 rpm.

The third block examined the combined effects of the individualized relative power and cadence. The relative power was set to 1.5 W/kg and 3 W/kg. During the warm-up, the cyclists rode for 8 min at 1.0–1.3 W/kg and 70–80 rpm, then for one minute at 1.5 W/kg and one minute at 3 W/kg using a freely chosen cadence. The natural cadence for each cyclist at each power level was defined as the mean cadence in the final 20 s of the warm-up intervals. Four experimental conditions were then completed: 1.5 W/kg at natural cadence, 1.5 W/kg at cadence reduced by 20%, 3 W/kg at natural cadence, and 3 W/kg at cadence reduced by 20%. Each condition lasted 30 s, with the last 6 s recorded, followed by 2 min of active recovery at warm-up intensity. Because cadence was individualized in this block, the number of pedal revolutions recorded during the 6 s window varied across cyclists and conditions, but the analysis was always performed during the final steady-state portion of each trial after the stabilization period.

The plantar surface was divided into nine anatomical regions arranged from medial to lateral and from forefoot to rearfoot: distal forefoot regions D1 (medial/distal), D2 (central/distal), and D3 (lateral/distal); central forefoot regions C1 (medial/central), C2 (central), and C3 (lateral/central); midfoot regions M1 (medial midfoot) and M2 (lateral midfoot); and T, corresponding to the posterior plantar support region. Thus, D1 and C1 represent the medial plantar forefoot, D3 and C3 the lateral plantar forefoot, D2 and C2 the central forefoot, M1 the medial midfoot, and M2 the lateral midfoot.

### 2.7. Statistical Analysis

All statistical analyses were performed using Statistica 8.0 (StatSoft Inc., Tulsa, OK, USA). Descriptive statistics are expressed as mean and standard error (SE). Normality was assessed using the Shapiro–Wilks test, and extreme outliers were removed following the recommendations of Field [28]. Although the variables were not normally distributed, parametric analyses were justified because of the robustness of ANOVA in non-normal conditions, the relative nature of normality in biomechanical datasets, and the statistical power of the design. In addition, residual distributions and model assumptions were inspected graphically before analysis, and homoscedasticity was verified for all variables. Homoscedasticity was verified for all variables. Sphericity was assessed using Mauchly’s test, and Greenhouse–Geisser or Huynh–Feldt corrections were applied when the assumption was violated. Partial eta squared (ηp^2^) was reported as the measure of effect size for all ANOVA models.

To evaluate the effects of absolute power, repeated-measures ANOVA was conducted with power (100 vs. 200 W), foot (left vs. right), and region (nine plantar regions) as within-subject factors. A parallel analysis was performed for absolute cadence using cadence (80 vs. 100 rpm), foot, and region. For the individualized block, a repeated-measures ANOVA was used to examine the effects of relative power (1.5 vs. 3 W/kg), cadence (natural vs. 20% reduced), foot, and region. Injury analyses used mixed repeated-measures ANOVA designs with the injury group (NP vs. each pathology) as the between-subject factor and the relevant within-subject factors from each block, using both the nine-region configuration and the pooled transverse and longitudinal segments. When significant or marginal effects were identified (*p* < 0.05 or *p* < 0.10), Bonferroni-adjusted pairwise comparisons were performed. Sex was not included as a covariate in the statistical models because only six women participated, all within the individualized protocol, which precluded robust covariate or subgroup analyses by sex. To examine pathology-specific patterns, separate mixed repeated-measures ANOVAs were performed for each injury category by comparing cyclists classified in that category with the NP group. Because some participants met the criteria for more than one injury, the pathology-specific analyses were conducted independently and not as a single mutually exclusive multi-group model. Accordingly, these comparisons should be interpreted as condition-specific exploratory analyses rather than fully independent between-group contrasts.

## 3. Results

**Figure 2:**
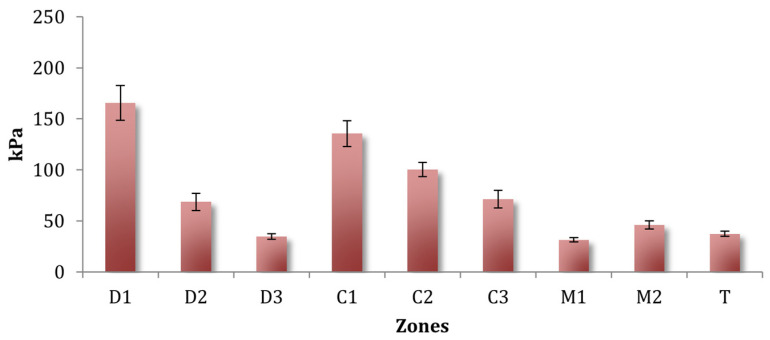
Mean ± SE of maximum plantar pressure across the nine plantar regions. D1 and C1 correspond to the medial forefoot regions, whereas D3 and C3 correspond to the lateral forefoot regions.

Because some participants were included in more than one pathology-specific category, the reported group effects did not fully reflect independent between-group comparisons. Accordingly, these findings should be interpreted as exploratory patterns rather than definitive differences between groups. Across all conditions, plantar pressure distribution showed a consistent spatial pattern dominated by the forefoot, particularly the medial and central regions (Table 2 and Table 3). In the absolute-load trials, maximal pressures differed significantly by plantar region, with the highest values repeatedly observed at the medial forefoot (D1 and C1) and progressively lower pressures toward the lateral and posterior areas (Figure 2). This regional effect was robust (*p* < 0.001) and was reproduced in all experimental blocks.

### 3.1. Effect of Increasing Power

**Figure 3:**
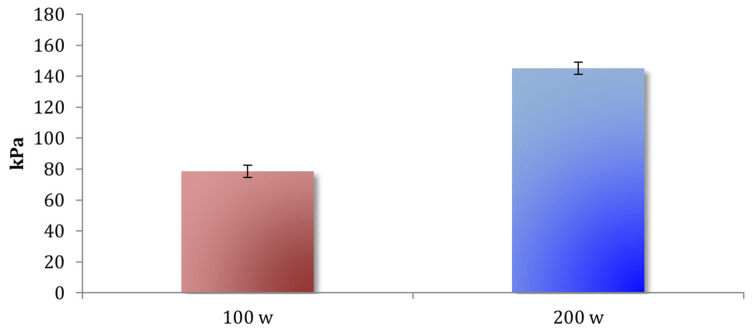
Mean ± SE of maximum plantar pressure at 100 W and 200 W during the absolute power protocol.

When cadence was held constant at 100 rpm, increasing the absolute power from 100 W to 200 W produced a strong rise in maximal plantar pressures across the foot (Table 4). A main effect of power was found, F(1,41) = 159.87, *p* < 0.001, ηp^2^ = 0.79, 95% CI (0.676, 0.862), Given the repeated-measures design and the strong within-subject contrast between load conditions, this large effect size indicates that pedaling power accounted for a substantial proportion of the explainable variance in maximal plantar pressure, with pressures 84.74% higher at 200 W than at 100 W (Figure 3). The regional pattern remained forefoot-dominant, with D1 and C1 presenting the highest pressures and no differences between them (*p* = 0.704), whereas medial regions were consistently higher than lateral ones (all *p* < 0.001). Power interacted with region, F(2.51,102.76) = 34.82, *p* < 0.001, ηp^2^ = 0.46, indicating a nonuniform amplification of pressure; the largest increases occurred in the medial forefoot zones (C1 +158%, D1 +96%), whereas the lateral regions showed smaller changes (C3 +28%, M2 +19%, D3 +13%). No main effect of the foot was detected (*p* = 0.933).

Given the limited sample sizes in some pathology-specific subgroups, particularly AT and ITBS, the following subgroup analyses should be interpreted cautiously, particularly for higher-order interactions. These findings are best considered preliminary and hypothesis-generating in nature.

### 3.2. Power Effects by Injury Group

**Figure A1:**
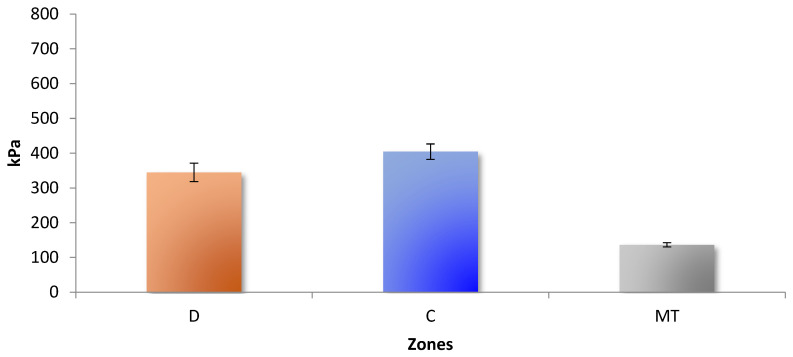
Average and Standard Error of the maximum pressure as a function of the Zone on the transverse segments.

**Figure A2:**
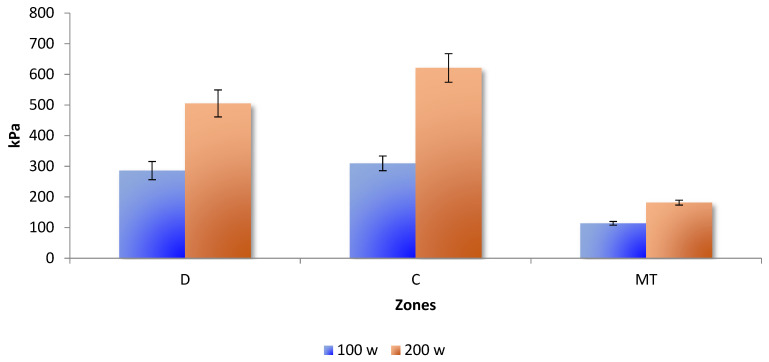
Average and Standard Error of the maximum pressure as a function of Zone and Power.

In the exploratory subgroup analysis, cyclists with Achilles tendinopathy tended to show higher maximal pressures than non-pathological cyclists when power was manipulated. An exploratory group effect was observed (F(1,26) = 4.48, *p* = 0.044, ηp^2^ = 0.15), suggesting that TA cyclists produced 27.44% greater pressures overall; however, this result should be interpreted cautiously, given the small subgroup size. The spatial distribution by transverse segments showed higher pressures in forefoot segments (D and C) than mid/rearfoot (MT), F(2,52) = 17.16, *p* < 0.001, ηp^2^ = 0.40 (Figure A1). Power increased pressure across all segments with a significant Power × Segment interaction, F(2,52) = 8.85, *p* < 0.001, ηp^2^ = 0.25 (Figure A2). A strongly medial loading dominance was confirmed in longitudinal segmentation, F(1,26) = 83.95, *p* < 0.001, ηp^2^ = 0.76, and power amplified medial pressures markedly more than lateral ones.

**Figure A3:**
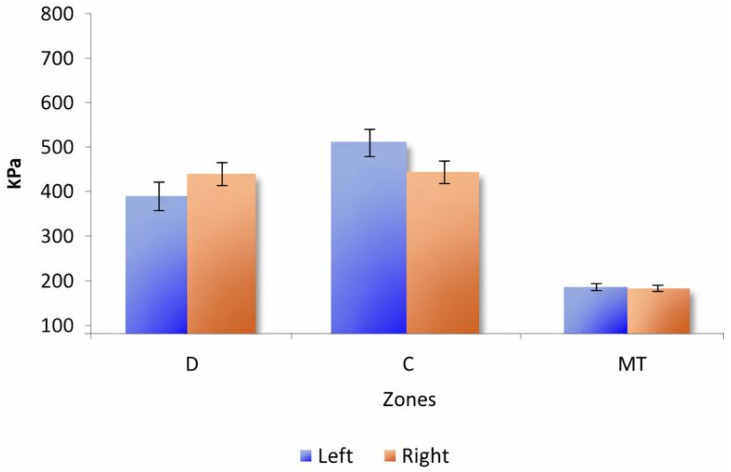
Average and Standard Error of the maximum pressure as a function of Zone and Foot.

**Figure A4:**
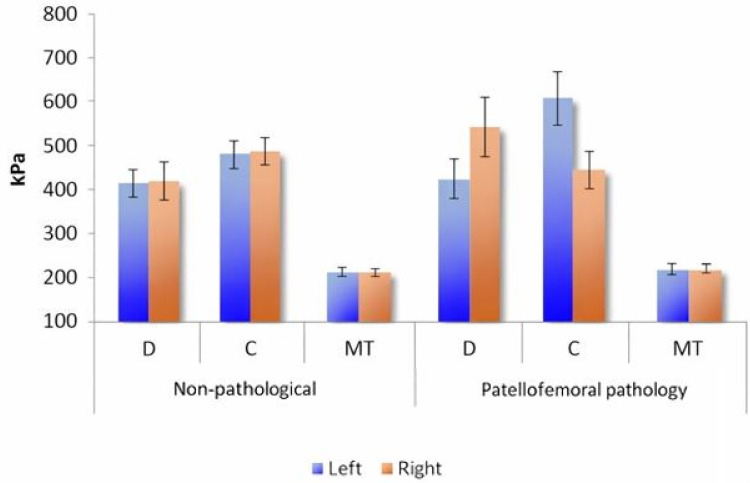
Average and Standard Error of maximum pressure according to foot and zone, in NP and PFP cyclists.

For patellofemoral pathology, the overall pressure magnitude did not differ from that of NP cyclists (group *p* = 0.181), whereas the within-subject power-related increase in pressure and forefoot-dominant pattern were reproduced. The key finding was altered right–left distribution. A Foot × Segment interaction was detected, F(1.50,46.66) = 5.42, *p* = 0.013, ηp^2^ = 0.15 (Figure A3), and a Group × Foot × Segment interaction, F(1.50,46.66) = 6.07, *p* = 0.009, ηp^2^ = 0.16, showing higher pressures in left-foot C and a tendency to higher right-foot D in PFP cyclists, while NP cyclists remained symmetrical (Figure A4).

**Figure A5:**
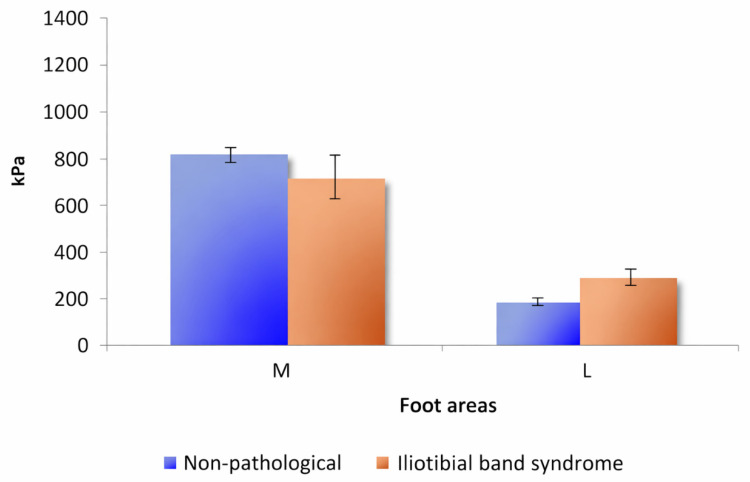
Pressure differences between non-pathological participants and those with ITBS in the longitudinal segment areas.

In cyclists with iliotibial band syndrome, total pressure did not differ from that of NP cyclists (group *p* = 0.862), whereas the within-subject effects of power were again observed. Medial–lateral analysis suggested a possible lateral bias: Group × Segment was marginal, F(1,25) = 3.15, *p* = 0.088, ηp^2^ = 0.11, with SCIT cyclists tending to produce 73.76% higher pressures laterally than NP cyclists (Figure A5). Given the limited size of this subgroup, this pattern should be interpreted as preliminary only.

### 3.3. Effect of Increasing Cadence

With absolute power fixed at 100 W, increasing the cadence from 80 rpm to 100 rpm increased the maximal pressures (Table 3). A main cadence effect was observed, F(1,41) = 10.08, *p* = 0.003, ηp^2^ = 0.20, with pressures 15.90% higher at 100 rpm. Pressures differed by region (F (2.91,119.40) = 30.91, *p* < 0.001, ηp^2^ = 0.43), peaking in the medial forefoot zones (Figure 2). Cadence interacted with region, F(2.65,108.61) = 3.30, *p* = 0.028, ηp^2^ = 0.07, showing the greatest cadence-related increases in D1 (+28.98%) and D2 (+23.98%) and minimal effects in the lateral zones. A Foot × Region interaction emerged, F(3.18,130.50) = 2.85, *p* = 0.037, ηp^2^ = 0.06, driven by higher left-foot pressure in C1.

### 3.4. Cadence Effects by Injury Group

**Figure A6:**
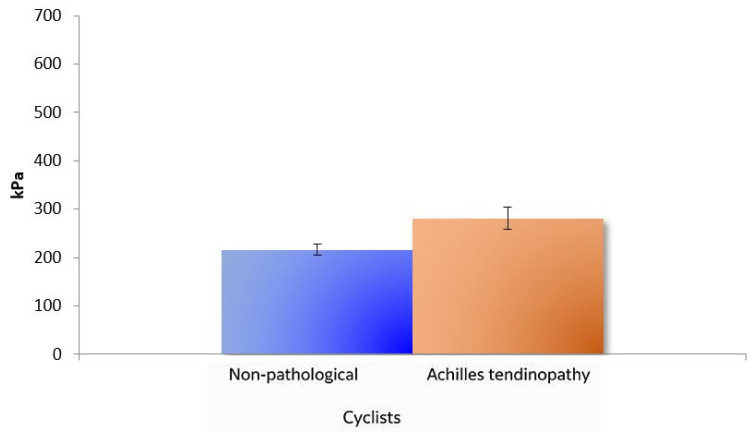
Average and Standard Error of maximum pressure in NP and AT participants (transverse analysis).

**Figure A7:**
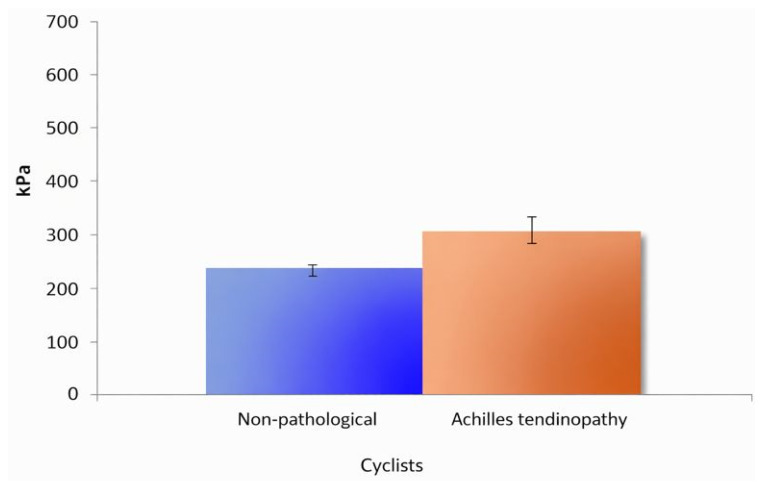
Average and Standard Error of maximum pressure in cyclists with AT and NP (longitudinal analysis).

In the exploratory cadence subgroup analysis, cyclists classified with Achilles tendinopathy again tended to show globally higher pressures than NP cyclists during cadence manipulation (Table 5). The group effect reached statistical significance in the transverse analysis, F(1,24) = 6.24, *p* = 0.020, ηp^2^ = 0.21 (Figure A6), and in the longitudinal analysis, F(1,24) = 6.51, *p* = 0.017, ηp^2^ = 0.21 (Figure A7); however, both findings should be interpreted cautiously because of small subgroup sizes. Cadence effects depended on foot side: Group × Foot × Cadence was significant, F(1,24) = 5.95, *p* = 0.022, ηp^2^ = 0.20, showing a stronger cadence increase in the left foot of TA cyclists. In longitudinal segmentation, cadence effects were mainly medial (Cadence × Segment: F(1,24) = 4.50, *p* = 0.044, ηp^2^ = 0.16).

**Figure A8:**
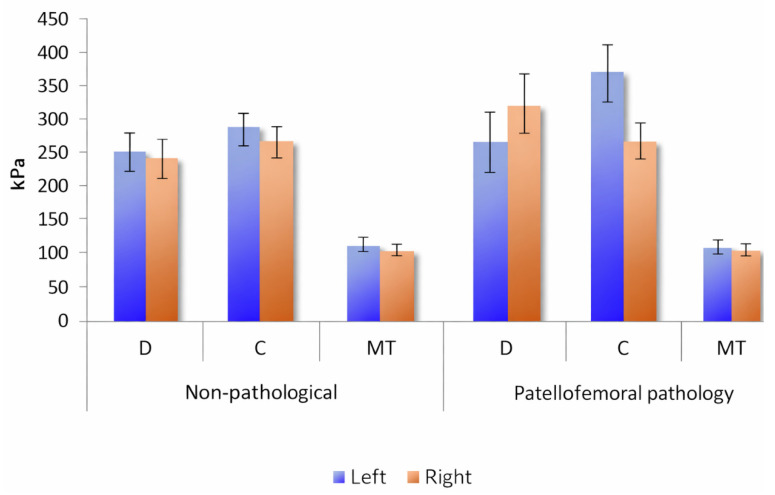
Average and Standard Error of the pressure differences between the left and right foot, in the areas of the transverse segments (D, C, MT) in NP and PFP cyclists.

For patellofemoral pathology, cadence increased pressures similarly to NP cyclists (group *p* = 0.210); however, a bilateral asymmetry pattern again emerged. Foot × Segment was significant, F(1.71,49.53) = 3.92, *p* = 0.032, ηp^2^ = 0.12, with higher left foot C pressures. Group × Foot × Segment showed a marginal trend (F(1.71,49.53) = 2.65, *p* = 0.088, ηp^2^ = 0.08; Figure A8). A marginal Group × Foot × Cadence interaction, F(1,29) = 4.04, *p* = 0.054, ηp^2^ = 0.12, suggested that cadence increased pressure in PF cyclists more than in NP cyclists.

In ITBS cyclists, cadence produced the expected within-subject increase and preserved forefoot dominance without differences between groups. Bilateral effects appeared mainly in longitudinal segmentation as Foot × Segment, F(1,24) = 6.24, *p* = 0.020, and ηp^2^ = 0.21.

#### Combined Individualized Effects of Relative Power and Individualized Cadence

**Figure 4:**
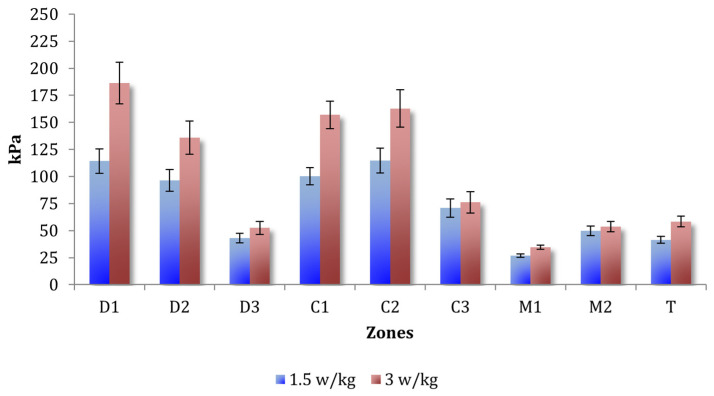
Mean ± SE of maximum plantar pressure by plantar region at 1.5 and 3.0 W·kg^−1^ during the individualized relative power protocol.

**Figure 5:**
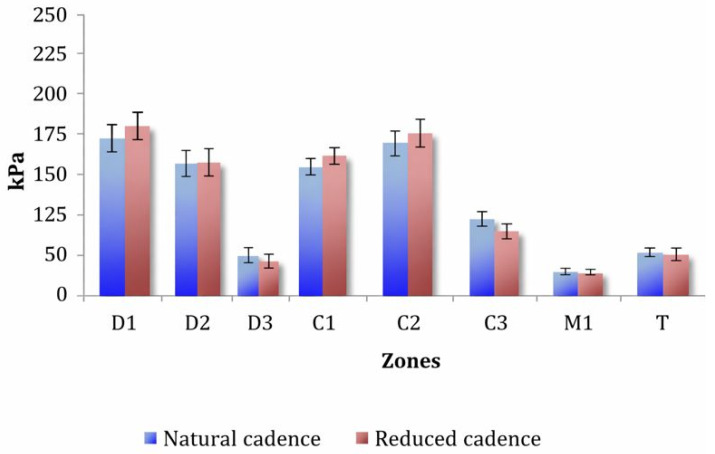
Mean ± SE of maximum plantar pressure by plantar region under natural cadence and cadence reduced by 20% during the individualized protocol.

**Figure A9:**
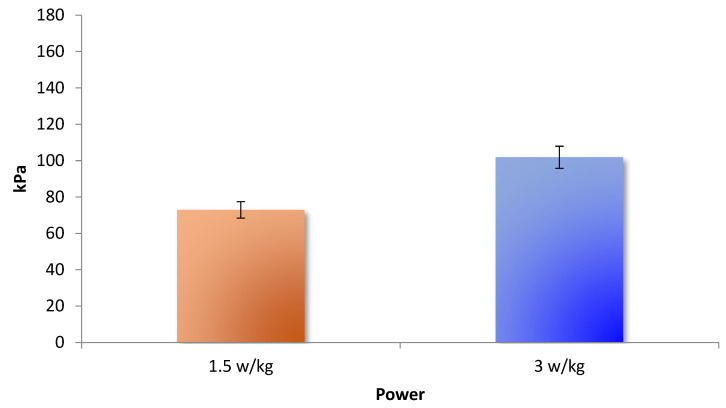
Average and Standard Error of maximum pressure as a function of pedaling power.

Under individualized loading, increasing the relative power from 1.5 W/kg to 3 W/kg produced another strong increase in plantar pressure (Table 2). A main power effect was observed, F(1,48) = 171.24, *p* < 0.001, ηp^2^ = 0.78, 95% CI (0.667, 0.847). This large effect size is consistent with the marked within-subject influence of relative power on plantar pressure observed across the repeated conditions, with pressures 39.59% higher at 3 W/kg (Figure A9). Region effects replicated, and relative power interacted with region, F(30.05,146.38) = 25.86, *p* < 0.001, ηp^2^ = 0.35 (Figure 4), again showing preferential medial amplification. Cadence had no global main effect (*p* = 0.309), but its influence varied regionally (Cadence × Region: F(4.49,215.54) = 6.04, *p* < 0.001, ηp^2^ = 0.11), increasing pressures in the medial forefoot areas while reducing them laterally (Figure 5). Small bilateral differences persisted depending on the region (Foot × Region: F(3.76,180.73) = 2.59, *p* = 0.041).

### 3.5. Combined Effects by Injury Group

**Figure 6:**
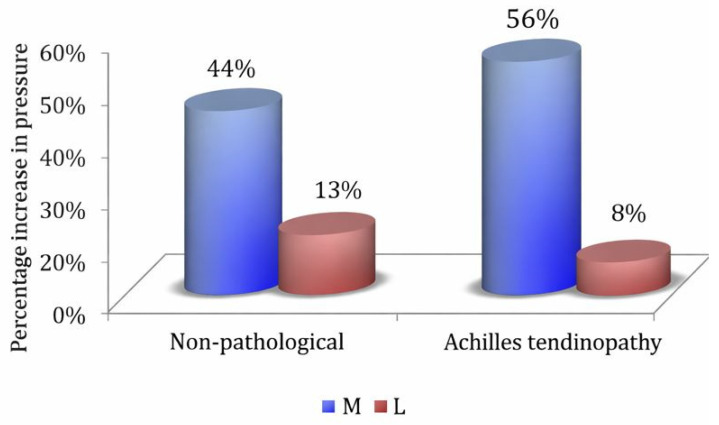
Percentage increase in maximum plantar pressure due to power output in the medial (M) and lateral (L) longitudinal segments in cyclists with AT and NP.

**Figure A10:**
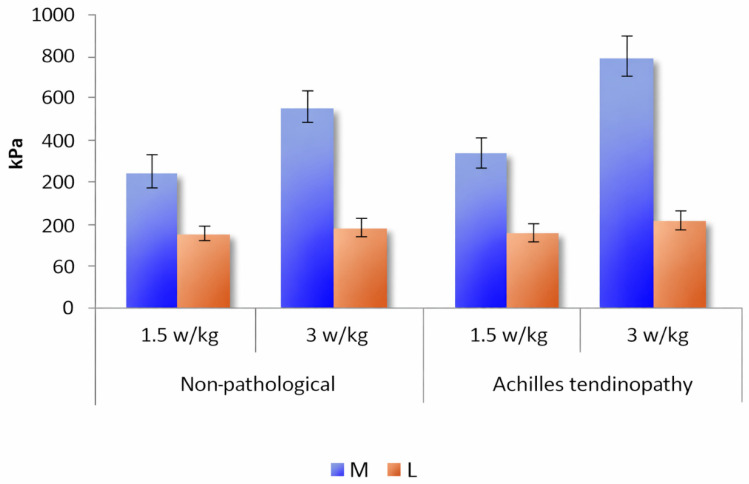
Average and Standard Error of maximum pressure as a function of Power and Zone in cyclists with AT and NP.

Under individualized conditions, cyclists classified with Achilles tendinopathy did not differ from NP cyclists in terms of overall pressure magnitude (group *p* = 0.537). However, exploratory differences in medial–lateral adaptation to increasing power were observed. Group × Power × Segment was significant, F(1,24) = 6.19, *p* = 0.020, ηp^2^ = 0.20 (Figure A10), showing that TA cyclists increased medial pressures more than NP cyclists with higher load, producing larger medial–lateral divergence (Figure 6).

**Figure 7:**
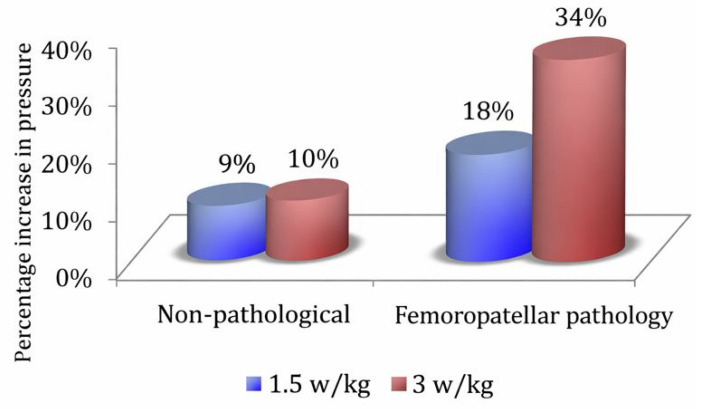
Percentage difference between both feet at different power outputs in cyclists with PFP and NP.

**Figure A11:**
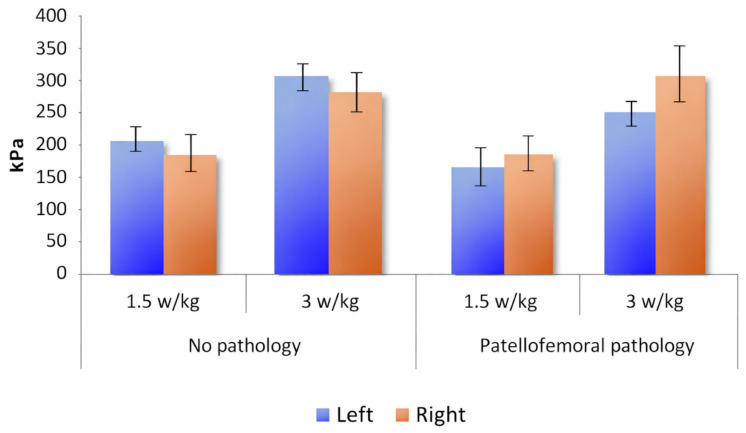
Average and Standard Error of maximum pressure as a function of Power and Foot in cyclists with PFP and NP.

In patellofemoral pathology, overall pressures did not differ from those of NP cyclists (group *p* = 0.372). An asymmetry pattern was observed: Group × Foot was significant, F(1,26) = 5.33, *p* = 0.029, and Group × Foot × Power was significant, F(1,26) = 5.17, *p* = 0.031 (Figure A11), indicating that PF cyclists loaded the right foot more and that this asymmetry enlarged at 3 W/kg (Figure 7).

**Figure 8:**
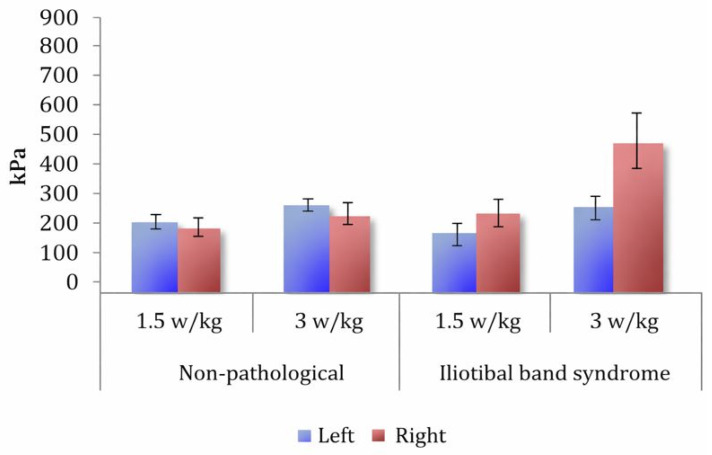
Mean and Standard Error of maximum pressure as a function of Power and Foot in NP cyclists and those with ITBS.

**Figure A12:**
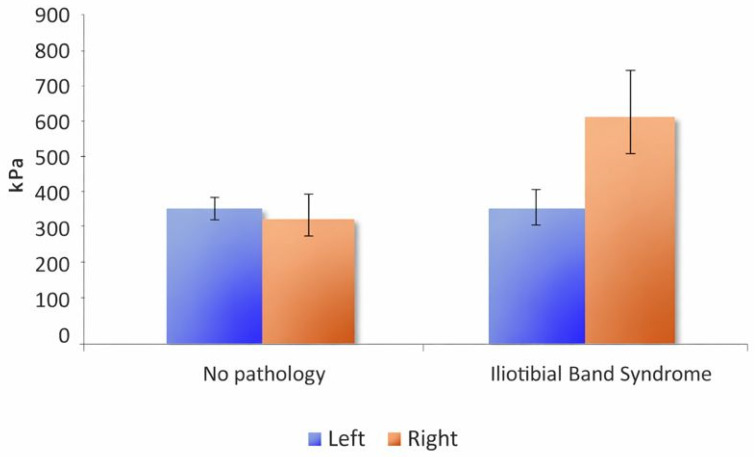
Average and Standard Error of maximum pressure as a function of the foot in NP cyclists and with ITBS.

**Figure A13:**
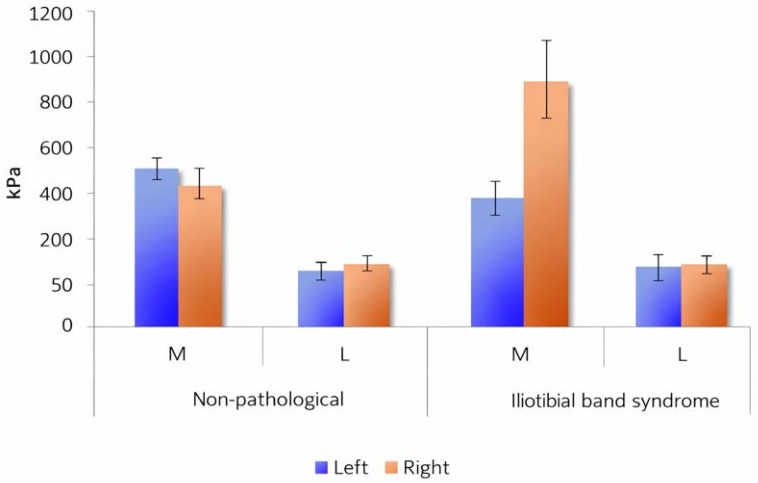
Average and Standard Error of maximum pressure as a function of Foot and Zone in NP and ITBS cyclists.

For ITBS cyclists, the total pressures were not different from those of NP cyclists (group *p* = 0.470), and cadence was not globally influential (*p* = 0.378). However, an apparent right–left asymmetry was observed in this subgroup. The Group × Foot interaction was significant, F(1,20) = 7.79, *p* = 0.011 (Figure A12), and the Group × Foot × Power interaction was significant, F(1,20) = 5.55, *p* = 0.029 (Figure 8), with ITBS cyclists producing much higher right-foot pressures at both loads. This right-foot dominance also appeared across the transverse segments and longitudinal segmentation (Figure A13), although this finding should be considered preliminary.

## 4. Discussion

This study investigated plantar pressure distribution in professional cyclists using a unified experimental protocol that manipulated pedaling power and cadence, while also exploring potential differences associated with overuse injuries. The strongest findings of this study arose from the within-subject effects of power and cadence, which were consistently observed across experimental conditions. By integrating absolute load, cadence manipulation, and individualized relative-load conditions, the design allowed us to characterize three core outcomes: the fundamental structure of plantar pressure distribution during cycling, mechanical modulation of that structure by power and cadence, and injury-specific deviations in spatial loading patterns and in bilateral symmetry.

### 4.1. Fundamental Pattern of Plantar Pressure Distribution

Across all experimental conditions, plantar loading demonstrated a stable organization characterized by dominant forefoot pressure, particularly in the medial and central regions. This pattern was consistently reproduced for all data blocks (Table 2). Maximal pressures were localized at the hallux (D1) and medial metatarsal head (C1), with a progressive decline toward the lateral forefoot and midfoot zones. This distribution aligns with prior cycling literature describing forefoot-centric pressure profiles [29,30], although our results did not reproduce the “metatarsal arch” load pattern (higher pressures at the fifth metatarsal) described in some earlier studies [29,30]. These differences may reflect sensor configuration variability [31], shoe–pedal interface differences, and the elite skill level of the participants. This forefoot-dominant pattern is mechanically consistent with the constrained shoe–pedal interface in cycling and the anterior location of force transmission; therefore, it should not be interpreted as a surprising finding. Rather, the relevance of the present results lies in quantifying how this baseline pattern is modulated by power, cadence, and injury status in elite cyclist populations.

The robustness of this spatial pattern across power, cadence, and individualized trials supports the conclusion that elite cyclists maintain a conservative pressure map driven by a consistent mechanical alignment of the lower limb and shoe–pedal interface.

#### 4.1.1. Effects of Increasing Power

Increasing pedaling power—whether applied absolutely (100–200 W) or relatively (1.5–3 W/kg)—produced the strongest and most uniform rise in plantar pressure across the foot. Absolute power manipulation yielded an 84.7% increase in peak pressure (Figure 3), whereas relative power increased the pressure by 39.6%. These findings support previous reports demonstrating that higher power increases pedal force, muscle activation, and joint loading [3,4,5].

Power-related increases were spatially non-uniform, with the medial forefoot zones (D1 and C1) showing the largest amplification. This partially aligns with the findings of Sanderson et al. [5], who reported preferential increases in the first-ray region when power increased [5]. This pattern likely reflects increased activation of the plantar flexors and a more anterior–medial force application strategy during high-demand pedaling [2,32].

#### 4.1.2. Effects of Increasing Cadence

When the absolute power was fixed at 100 W, increasing the cadence from 80 to 100 rpm produced a moderate but significant increase in plantar pressure, driven primarily by increases at D1 and D2. Although this finding contrasts with the classical model in which cadence reduces plantar pressure [5,9], it matches the exact low-power/high-cadence condition reported by Sanderson et al. [5]. This supports the notion that cadence effects depend critically on the interaction between cadence and power, not cadence alone.

Under individualized conditions, cadence did not produce a global main effect, but systematically redistributed pressure medially while decreasing lateral loading. This redistribution may be related to postural stabilization demands during high-spin, low-load pedaling, as previously suggested by Sanderson et al. [5].

#### 4.1.3. Exploratory Injury-Related Biomechanical Patterns

Because no kinematic, electromyographic, or joint angle data were collected, the mechanistic interpretations discussed below should be regarded as plausible hypotheses based on the observed plantar pressure patterns rather than as direct causal explanations.

#### 4.1.4. Achilles Tendinopathy (AT)

Cyclists classified with AT tended to demonstrate higher pressures than non-injured cyclists under fixed load and cadence conditions. In addition, AT cyclists showed a distinctive medial amplification pattern with rising power under individualized conditions, as reflected in a significant Group × Power × Segment interaction; however, this pattern should be regarded as preliminary and requires further investigation. This aligns with studies linking TA to increased forefoot and hallux loading [15,33,34] and may reflect heightened gastrocnemius–soleus recruitment or bike-fit factors, such as excessive plantarflexion or dorsiflexion angles, although these mechanisms were not directly assessed in the present study [35].

#### 4.1.5. Patellofemoral Pain (PFP)

PFP cyclists did not demonstrate globally elevated pressures but showed a recurring right–left asymmetry pattern, especially under higher loads. This suggests that PFP is consistent with the possibility of compensatory offloading or altered motor patterns rather than simple pressure magnitude; however, these mechanisms cannot be confirmed without concurrent kinematic or neuromuscular measurements. Similar medial–lateral deviations have been reported in running and walking in PF subjects [16], although cycling-specific findings are scarce.

#### 4.1.6. Iliotibial Band Syndrome (ITBS)

The ITBS cyclists appeared to exhibit the strongest asymmetry pattern. Even when the total pressures matched the controls, ITBS cyclists appeared to load the right foot more (Figure 8). Medial pressure amplification with increasing power may be compatible with the proposed biomechanical mechanisms involving excessive internal rotation or pronation, increasing iliotibial band friction [36,37]. This medial loading phenotype contrasts with the lateral overload patterns described in other contexts and may reflect the heterogeneity of ITBS mechanisms; however, this interpretation remains uncertain.

Taken together, the most robust contribution of the present study is the demonstration that plantar pressure distribution in elite cyclists is strongly and consistently modulated by pedaling power, whereas cadence exerts a more modest but region-specific effect. Pathology-specific analyses should be viewed as exploratory extensions of these core within-subject findings.

#### 4.1.7. Limitations

Several limitations must be considered when interpreting the findings of this study. First, the cross-sectional and observational nature of the study prevents the establishment of causal relationships between plantar pressure patterns and overuse injuries; the results identify associations, not mechanisms of injury development. Second, although the overall sample size was large for elite cycling research, the number of cyclists within some injury groups, particularly Achilles tendinopathy, was relatively small. This inevitably reduces the statistical power for between-group comparisons and increases the probability of Type II errors. In addition, some pathology-specific subgroup analyses may have been underpowered and should therefore be interpreted as exploratory. Third, the experimental protocol was conducted in a controlled laboratory environment using stationary trainers. While standardization is essential for isolating the effects of power and cadence, indoor pedaling does not perfectly reproduce outdoor kinetics, environmental variability, or neuromuscular adaptations that occur during real competition. Fourth, because injuries were assessed at a single time point, the temporal relationship between plantar pressure patterns and injuries cannot be established. Therefore, it is not possible to determine whether the observed alterations in plantar pressure preceded the development of the injury, represented compensatory adaptations to existing pathology, or reflected long-standing motor patterns. Longitudinal studies are required to clarify the directionality and causal mechanisms underlying these associations.

An important limitation is that some cyclists met the criteria for more than one overuse injury and were therefore included in multiple pathology-specific analyses. This overlap violates the statistical independence of the comparison groups and limits the interpretability of the between-group effects. Consequently, the reported pathology-specific differences should not be interpreted as true independent group contrasts but rather as exploratory patterns that may partially reflect shared participant characteristics. Future studies should use mutually exclusive group designs or larger samples to allow for fully independent comparisons. Another limitation was that the pedal–cleat systems were not standardized across participants. Differences in pedal platform geometry, cleat design, and degree of angular float may influence how force is transmitted to the plantar surface, and may therefore modify both the magnitude and regional distribution of plantar pressure. Although the cyclists used their habitual competition setups to enhance ecological validity, the potential effect of different pedal systems on plantar loading cannot be excluded from consideration.

Similarly, the cycling shoe type was not standardized or formally monitored in this study. Differences in shoe construction, sole stiffness, fit, and the shoe–cleat interface may alter the plantar support, local deformation, and pressure dispersion across the forefoot and midfoot. Thus, some of the observed inter-individual variability in plantar pressure distribution may have been influenced by footwear characteristics, in addition to biomechanical factors.

In addition, the cleat position and angular float were not standardized or formally monitored. Because cleat setback, mediolateral placement, and rotational freedom may affect foot posture, force application, and direction of load transfer during pedaling, these factors may also have contributed to the observed pressure patterns. Furthermore, pathology-specific subgroup findings should be interpreted as preliminary and hypothesis-generating. The small numbers in some injury categories, particularly AT and ITBS, limit the stability of subgroup estimates and reduce the confidence in higher-order interactions. These analyses are useful for identifying potentially meaningful biomechanical patterns; however, they should not be interpreted as definitive evidence of injury-specific plantar pressure signatures until confirmed in larger independent samples.

Finally, although the individualized protocol included both men and women, only six women were enrolled, and their data were pooled with those of the male cyclists. Consequently, this study was not designed or powered to examine sex-specific plantar pressure responses, and the generalizability of the findings to female professional cyclists remains limited.

## 5. Conclusions

Professional cyclists exhibit a characteristic medial forefoot-dominant plantar pressure profile that is consistently modulated by pedaling power and, to a lesser extent, by the cadence. Power emerged as the clearest determinant of plantar pressure magnitude, whereas cadence mainly influenced regional load distribution depending on the test condition. These within-subject findings represent the most robust contribution of this study. Pathology-specific analyses suggest that overuse injuries may be associated with distinct asymmetry and loading patterns; however, these subgroup findings are preliminary, exploratory, and hypothesis-generating because of overlapping classifications and limited sample sizes in some injury categories. Future studies with larger and fully independent injury groups are required to confirm these potential injury-specific plantar pressure signatures.

## Figures and Tables

**Table 1:** Distribution of participants according to overuse injury classification, including overlapping injury categories.

Type of Injury	No. of Participants
Achilles tendinopathy (AT)	7
Patellofemoral pathology (PFP)	10
Iliotibial band syndrome (ITBS)	6
Others (OT)	8
Non-pathological (NP)	27
TOTAL (*n*)	50

Note: The total number of injury classifications exceeds the number of participants because 8 cyclists presented with more than one overuse injury and were therefore included in multiple pathology categories. These groups are not mutually exclusive.

**Table 2:** Mean (±SE) maximum plantar pressure by plantar region, foot, relative power output, and individualized cadence condition.

	1.5 w/kg				3 w/kg			
	Natural Cad.		Reduced Cad.		Natural Cad.		Reduced Cad.	
	Right Foot	Left Foot	Right Foot	Left Foot	Right Foot	Left Foot	Right Foot	Left Foot
D1	106.62 (±14.23)	116.16 (±14.96)	112.19 (±14.12)	191.16 (±28.67)	173.34 (±24.38)	185.74 (±22.98)	191.16 (±28.67)	194.99 (±25.76)
D2	106.87 (±15.53)	84.57 (±9.00)	113.01 (±17.57)	167.17 (±29.69)	160.23 (±24.35)	110.42 (±11.53)	167.17 (±29.69)	105.72 (±10.18)
D3	49.63 (±6.14)	40.01 (±4.40)	46.25 (±5.71)	59.39 (±8.35)	63.86 (±8.95)	44.64 (±6.05)	59.39 (±8.35)	41.96 (±5.55)
C1	80.28 (±8.24)	108.28 (±12.30)	88.16 (±9.16)	135.92 (±13.54)	123.89 (±12.33)	177.54 (±20.36)	135.92 (±13.54)	190.51 (±20.94)
C2	111.82 (±15.25)	108.87 (±11.50)	123.74 (±17.60)	184.78 (±28.86)	169.22 (±24.31)	144.45 (±15.75)	184.78 (±28.86)	152.65 (±13.95)
C3	83.14 (±12.18)	70.21 (±8.60)	72.24 (±11.38)	82.92 (±12.46)	95.03 (±17.24)	66.27 (±8.91)	82.92 (±12.46)	59.94 (±7.34)
M1	24.59 (±1.51)	27.09 (±1.88)	26.08 (±1.54)	33.04 (±2.53)	35.74 (±4.13)	34.57 (±2.75)	33.04 (±2.53)	34.91 (±2.58)
M2	54.55 (±6.26)	50.93 (±5.78)	46.34 (±3.75)	53.90 (±4.90)	58.40 (±6.12)	51.87 (±6.38)	53.90 (±4.90)	50.76 (±6.36)
T	41.63 (±4.24)	39.93 (±4.27)	44.18 (±4.58)	62.51 (±7.41)	61.67 (±7.37)	55.60 (±7.34)	62.51 (±7.41)	53.53 (±6.02)

Note: Natural cad. = cyclist’s natural cadence; reduced cad. = cadence reduced by 20% compared to natural. Plantar regions were coded as follows: D1 = medial distal forefoot, D2 = central distal forefoot, D3 = lateral distal forefoot; C1 = medial central forefoot, C2 = central forefoot, C3 = lateral central forefoot; M1 = medial midfoot, M2 = lateral midfoot; T = posterior plantar region.

**Table 3:** Mean (±SE) maximum plantar pressure by plantar region, foot, and cadence condition during the absolute cadence protocol.

	80 rpm		100 rpm	
	Right Foot	Left Foot	Right Foot	Left Foot
D1	146.94 (±20.74)	146.94 (±20.74)	195.71 (±25.66)	177.38 (±25.60)
D2	60.77 (±7.03)	60.77 (±7.03)	79.80 (±13.85)	71.99 (±8.33)
D3	34.33 (±3.05)	34.33 (±3.05)	37.64 (±3.31)	34.35 (±3.25)
C1	103.92 (±14.16)	103.92 (±14.16)	118.07 (±14.00)	164.95 (±18.17)
C2	90.09 (±7.14)	90.09 (±7.14)	103.10 (±8.76)	109.74 (±9.66)
C3	79.10 (±12.83)	79.10 (±12.83)	100.13 (±20.46)	85.18 (±11.84)
M1	28.89 (±2.04)	28.89 (±2.04)	53.02 (±5.45)	58.11 (±6.48)
M2	52.26 (±5.05)	52.26 (±5.05)	57.41 (±6.54)	54.50 (±7.75)
T	36.37 (±2.67)	36.37 (±2.67)	66.58 (±5.73)	68.37 (±5.36)

Note: Plantar regions were coded as follows: D1 = medial distal forefoot, D2 = central distal forefoot, D3 = lateral distal forefoot; C1 = medial central forefoot, C2 = central forefoot, C3 = lateral central forefoot; M1 = medial midfoot, M2 = lateral midfoot; T = posterior plantar region.

**Table 4:** Mean (±SE) maximum plantar pressure by plantar region, foot, and power condition during the absolute power protocol.

	100 w		200 w	
	Right Foot	Left Foot	Right Foot	Left Foot
D1	184.37 (±25.57)	163.55 (±25.50)	361.91 (±48.03)	319.07 (±29.05)
D2	78.30 (±13.71)	68.98 (±8.27)	125.12 (±15.53)	121.57 (±13.00)
D3	37.53 (±3.30)	34.04 (±3.29)	40.53 (±3.77)	40.55 (±3.22)
C1	119.56 (±13.91)	152.09 (±17.97)	321.65 (±35.71)	380.53 (±43.16)
C2	96.65 (±7.89)	98.19 (±8.78)	177.31 (±13.66)	180.03 (±18.26)
C3	79.10 (±12.83)	65.81 (±6.66)	100.13 (±20.46)	85.18 (±11.84)
M1	28.89 (±2.04)	34.31 (±4.52)	53.02 (±5.45)	58.11 (±6.48)
M1	52.26 (±5.05)	41.66 (±4.72)	57.41 (±6.54)	54.50 (±7.75)
T	36.37 (±2.67)	42.03 (±3.63)	66.58 (±5.73)	68.37 (±5.36)

Note: Plantar regions were coded as follows: D1 = medial distal forefoot, D2 = central distal forefoot, D3 = lateral distal forefoot; C1 = medial central forefoot, C2 = central forefoot, C3 = lateral central forefoot; M1 = medial midfoot, M2 = lateral midfoot; T = posterior plantar region.

**Table 5:** Mean (±SE) maximum plantar pressure in transverse plantar segments in non-pathological cyclists and cyclists with Achilles tendinopathy during the cadence protocol.

	Non-Pathological (*n* = 27)				Achilles Tendinopathy (*n* = 7)			
	80 rpm		100 rpm		80 rpm		100 rpm	
	Right Foot	Left Foot	Right Foot	Left Foot	Right Foot	Left Foot	Right Foot	Left Foot
D	214.14 (±35.36)	254.02 (±32.44)	266.34 (±51.15)	251.17 (±32.30)	328.40 (±72.46)	281.60 (±66.47)	437.70 (±104.82)	418.00 (±66.20)
C	261.99 (±32.99)	302.35 (±37.40)	288.95 (±28.92)	304.21 (±37.83)	355.02 (±67.60)	293.68 (±76.66)	379.88 (±59.28)	357.00 (±77.53)
MT	96.39 (±6.18)	113.12 (±8.56)	118.90 (±6.73)	116.71 (±11.68)	136.18 (±12.67)	120.44 (±17.54)	120.26 (±13.79)	146.88 (±23.95)

## Data Availability

The original contributions presented in this study are included in the article. Further inquiries can be directed to the corresponding author.
